# Inflammatory and oncogenic roles of a tumor stem cell marker doublecortin-like kinase (DCLK1) in virus-induced chronic liver diseases

**DOI:** 10.18632/oncotarget.3972

**Published:** 2015-04-29

**Authors:** Naushad Ali, Parthasarathy Chandrakesan, Charles B. Nguyen, Sanam Husain, Allison F. Gillaspy, Mark Huycke, William L. Berry, Randal May, Dongfeng Qu, Nathaniel Weygant, Sripathi M. Sureban, Michael S. Bronze, Danny N. Dhanasekaran, Courtney W. Houchen

**Affiliations:** ^1^ Department of Internal Medicine, Section of Digestive Diseases and Nutrition, University of Oklahoma Health Sciences Center, Oklahoma, OK, USA; ^2^ Infectious Diseases, University of Oklahoma Health Sciences Center, Oklahoma, OK, USA; ^3^ Department of Cell Biology, University of Oklahoma Health Sciences Center, Oklahoma, OK, USA; ^4^ Department of Pathology, University of Oklahoma Health Sciences Center, Oklahoma, OK, USA; ^5^ Department of Microbiology and Immunology, University of Oklahoma Health Sciences Center, Oklahoma, OK, USA; ^6^ Peggy and Charles Stephenson Cancer Center, University of Oklahoma Health Sciences Center, Oklahoma, OK, USA; ^7^ Department of Veterans Affairs Medical Center, University of Oklahoma Health Sciences Center, Oklahoma, OK, USA; ^8^ COARE Biotechnology, Oklahoma City, Oklahoma, OK, USA

**Keywords:** DCLK1, HCV, cancer stem cell, inflammation, hepatocellular carcinoma

## Abstract

Hepatocellular carcinoma (HCC) is the third most common cause of cancer-related mortality worldwide. We previously showed that a tumor/cancer stem cell (CSC) marker, doublecortin-like kinase (DCLK1) positively regulates hepatitis C virus (HCV) replication, and promotes tumor growth in colon and pancreas. Here, we employed transcriptome analysis, RNA interference, tumor xenografts, patient's liver tissues and hepatospheroids to investigate DCLK1-regulated inflammation and tumorigenesis in the liver. Our studies unveiled novel DCLK1-controlled feed-forward signaling cascades involving calprotectin subunit S100A9 and NFκB activation as a driver of inflammation. Validation of transcriptome data suggests that DCLK1 co-expression with HCV induces BRM/SMARCA2 of SW1/SNF1 chromatin remodeling complexes. Frequently observed lymphoid aggregates including hepatic epithelial and stromal cells of internodular septa extensively express DCLK1 and S100A9. The DCLK1 overexpression also correlates with increased levels of S100A9, c-Myc, and BRM levels in HCV/HBV-positive patients with cirrhosis and HCC. DCLK1 silencing inhibits S100A9 expression and hepatoma cell migration. Normal human hepatocytes (NHH)-derived spheroids exhibit CSC properties. These results provide new insights into the molecular mechanism of the hepatitis B/C-virus induced liver inflammation and tumorigenesis via DCLK1-controlled networks. Thus, DCLK1 appears to be a novel therapeutic target for the treatment of inflammatory diseases and HCC.

## INTRODUCTION

Hepatocellular carcinoma (HCC) has been recognized as the third most common cause of cancer-related mortality worldwide with an annual death toll of approximately 700,000 [1, 2]. According to World Health Organization, the global burden of HCC is expected to rise and will likely account for the second highest increase in cancer-related death rates by 2030 [3]. HCC often occurs in the setting of underlying chronic viral hepatitis, inflammation, steatosis, and cirrhosis. Chronic hepatitis C virus (HCV) infection is considered a major risk factor for the development of HCC. HCV causes chronic hepatitis in most patients (>80%), accounts for 50–76% of all primary liver cancer cases [4, 5] and reduces overall life expectancy by 8-12 years [6–8]. The risk for HCC increases in HCV carriers with obesity or diabetes, two prevalent diseases in the United States associated with chronic inflammation [1]. New direct-acting anti-HCV drugs against three key nonstructural proteins NS3, NS5A and NS5B have improved the treatment efficacy and cure rate of chronic HCV infection [9, 10]. However, the high costs of treatment [11], late stage detection of HCC, and co-existing cirrhosis pose major challenges for treating advanced disease. Epigenetic abnormalities and signaling dysregulation have also been shown to contribute to HCC [12]. Although chronic HCV infection is a major risk factor for HCC, how this virus directly contributes to hepatocellular transformation remains poorly understood.

CSCs display several characteristics of normal stem cells such as self-renewal, and unlimited proliferative and differentiation capacities [13]. These rare cells (1%–2% of total tumor mass) recapitulate the original tumor heterogeneity after metastasis and also contribute to cancer progression, metastasis and therapeutic resistance. Thus, CSCs represent translationally relevant targets for cancer treatment especially in malignancies that are refractory to conventional antitumor agents [14]. Histopathological study of tissues from chronic liver diseases, *in vitro* experiments and murine models support the existence of CSCs in HCC [reviewed in [12, 15]].

The doublecortin-like kinase 1 (DCLK1, domains organization shown in Figure S1) is a microtubule-associated CSC protein that catalyzes tubulin polymerization into microtubules. We previously demonstrated that DCLK1 is overexpressed in a number of solid tumors (colon, intestine, pancreas) including HCC [16–19]. Subsequently, our studies defined a role for DCLK1 in tumorigenesis and the activation of quiescent intestinal stem cells following radiation injury [18, 20, 21]. We also demonstrated that HCV replication positively correlates with several CSC-related proteins such as DCLK1, CD133, Lgr5, Lin28, AFP, CK19 and c-Myc [16]. siRNA knockdown of DCLK1 leads to diminished HCV replication [16] and downregulation of epithelial-mesenchymal transition (EMT)-promoting factors [17, 18]. Other investigators used lineage tracing in *Apc*^Min/+^ mice to demonstrate that DCLK1, in fact, marks CSCs in intestinal adenomas and its ablation results in regression of polyps [22]. These observations supported a potential association between DCLK1 and HCV-induced HCC.

Chronic inflammation associated with HCV infection is considered a major contributor to cirrhosis and the development of HCC. For example, inflammation shifts hepatocytic TGF-β signaling from tumor-suppression to fibrogenesis and an increased risk for HCC [23]. HCV infection also promotes production of the pro-inflammatory cytokine Interleukin-1β (IL-1β) through a caspase-1/NLRP3 inflammasome [24, 25]. The pro-inflammatory nuclear factor NFκB and calprotectin (S100A8/A9 heterodimer) are activated by HCV leading to induction of reactive oxygen species (ROS) and enhanced cell survival [26]. The S100A8 (myeloid related protein 8, MRP8) and S100A9 (MRP14) proteins belong to the S100 family of calcium-binding proteins. These molecules preferentially form an S100A8/A9 heterodimer in the presence of Zn^2+^ and Ca^2+^ that promotes phagocyte migration through tubulin polymerization and stabilization [27]. These proteins are constitutively expressed in myeloid cells in a calcium-dependent manner as multimers. S100A9 is an endogenous ligand for TLR4, RAGE (receptor for advanced glycation) and heparan sulfate. Increased expression of S100A8/A9 is reported in inflammatory diseases, cancer, cardiovascular disease, and autoimmunity [28–32]. Interestingly, DCLK1 binds and polymerizes tubulins into microtubules filaments [33, 34]. These observations indicate a putative functional relationship between DCLK1 and S100A8/9 via microtubule organization.

The DCLK1 gene functions in liver homeostasis or hepatic diseases are unclear. Here, we report the DCLK1's inflammatory and tumorigenic role in hepatitis B/C-induced cirrhosis and HCC. Our results further revealed plasticity of DCLK1-positive human hepatocytes and potential dedifferentiate into cancer-stem-like phenotypes. These cellular alterations are likely to drive progression of liver diseases into pre-neoplastic conditions such as fibrosis and cirrhosis and/or HCC.

## RESULTS

### Transcriptome analysis revealed a unique gene signature in DCLK1-overexpressing hepatoma cells that harbor HCV subgenomic replicons

**Figure 1:**
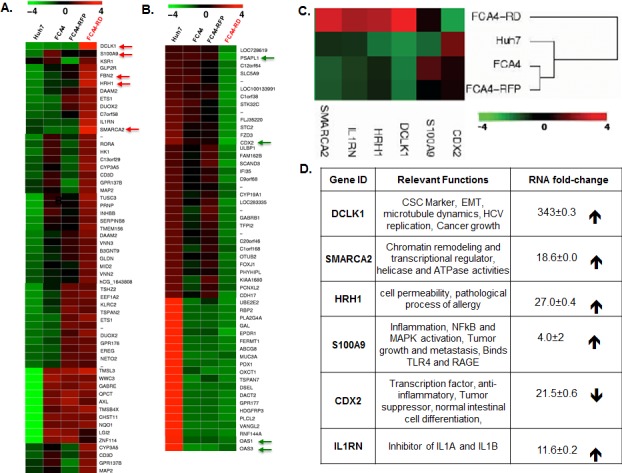

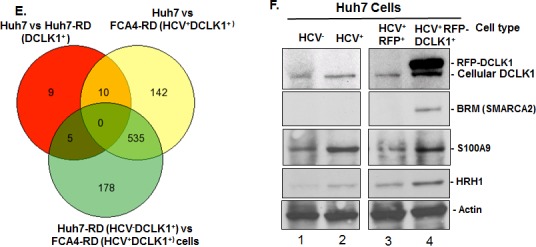
Differential gene expression profile in response to DCLK1 overexpression in the presence or absence of HCV The total RNAs isolated from Huh7 hepatoma cells expressing HCV subgenomic replicon (FCA4, HCV^+^) and FCA4 cells overexpressing recombinant RFP-DCLK1 (FCA4-RD, HCV^+^DCLK1^+^) were subjected to transcriptome analysis by RNASeq method. The data were analyzed using GeneSifter software at 10-fold change in the expression level and at least 10 reads (Quality). The results are derived from two independent biological and sequencing replicates. The FCA4-RFP (FCA4 expressing GFP), Huh7 and FAC4 were used as controls for the comparison of FCA4-RD transcriptome. **A.** and **B.** Heat-maps for differential gene expression in FCA4-RD cells. **C.** Cluster analysis for the selected genes. **D.** The reported gene functions and fold-changes in FCA4-RD RNAs with standard deviations as compare to the controls cell lines. **E.** Venn diagram showing total number of common and differentially regulated genes as indicated. **F.** Validation of three differentially regulated gene products by Western blot using cell lysates (30 μg). HCV and DCLK1 co-expression induces BRM/SMARCA2 expression and inflammatory molecules (S100A9 and HRH1).

The FCA4 cell line is derived from Huh7 hepatoma cells that persistently express G418-resistant subgenomic HCV-1b replicons [35, 36]. Staining for viral proteins suggest that the majority of cells express the replicon. The FCA4-derived FCA4-RD cells constitutively express human DCLK1 tagged at the N-terminus with red florescence protein (RFP-DCLK1, Figure S1). FCA4 cells expressing untagged RFP (FCA4-RFP) were used as controls for FCA4-RD cells. These cells were enriched for RFP-DCLK1 or RFP expression by FACS and used in the subsequent studies. Thus, most FCA4-RD cells represent HCV^+^DCLK1^+^ phenotypes. Total RNAs isolated from these cells were subjected to transcriptome analysis. As expected, the FCA4-RD cell line displayed overexpressed DCLK1 mRNAs (>300 fold) as compared to the parent (Huh7, FCA4) and control (FCA4-RFP) cell lines (Figures 1A and 1D). The heat maps of differentially regulated genes suggest that FCA4-RD expresses unique gene signatures characterized by enhanced expression of S100A9, GLP2R, FBN2, HRH1, IL1RN and SMARCA2 (human BRM) (Figures 1A and 1C). These genes are each involved in key inflammatory and carcinogenic pathways (Figure 1D). Nearly 30 genes, including PSAPL1 and CDX2, were downregulated in FCA4-RD cells compared to controls (Figure 1B). As expected, OAS1 and OAS3 that induce the degradation of viral RNAs in an interferon–dependent manner were downregulated in all three HCV replicon-expressing cell lines compared to HCV-negative Huh7 cells (Figure 1B, bottom arrows). During these analyses, we also addressed whether DCLK1 alone causes distinct regulation of gene expression by comparing transcriptomes of Huh7-RD cells, which express N-terminus RFP-tagged DCLK1 with that of parent Huh7 cells. In addition to an array of differentially expressed genes, Huh7-RD cells showed significant increases in S100A9 mRNA level (18.29±0.0002, Table S1). However, RFP expression alone in Huh7 cells failed to alter the S100A9 levels (not shown).

Gene ontology indicates that most of these differentially regulated genes in FCA4-RD cells potentially affect cellular and metabolic processes or signal transduction pathways (Figure S2). Comparative analyses of all cell lines clearly suggest that the highest number of altered gene expression was observed when the HCV replicon and recombinant DCLK1 were co-expressed (HCV^+^DCLK1^+^ cells). This result was obtained by analysis of mRNA levels in all the cell lines using Venn diagram (Figure 1E). In total, 142 to 178 genes showed unique changes in FCA4-RD cells. We validated some of these changes by Western blot (Figure 1F). Of note, the SMARCA2 (BRM) protein, an ATPase subunit of chromatin remodeling complex SW1/SNF1, was detected only in FCA4-RD cells (lane 4), and HRH1 was highest in these cells as compared to the controls.

### DCLK1 overexpression correlates with enhanced levels of S100A9, c-Myc, and SMARCA2 in patients

**Figure 2:**
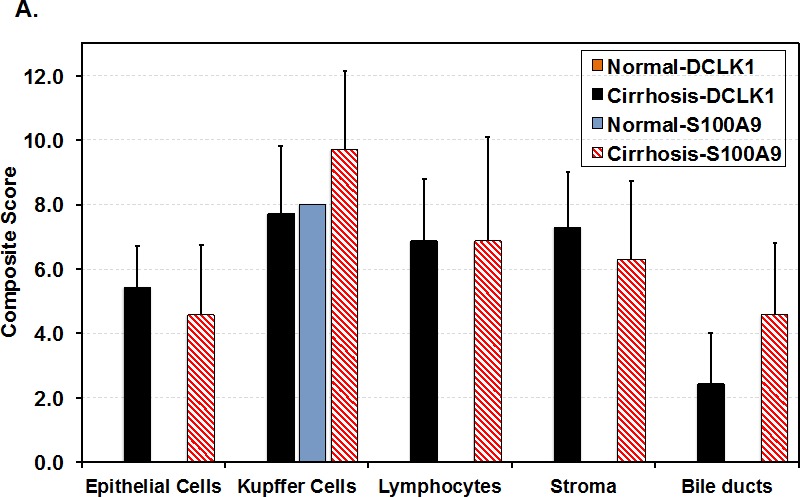

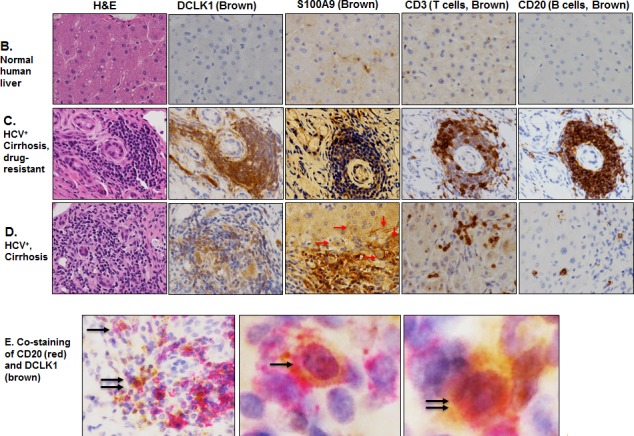

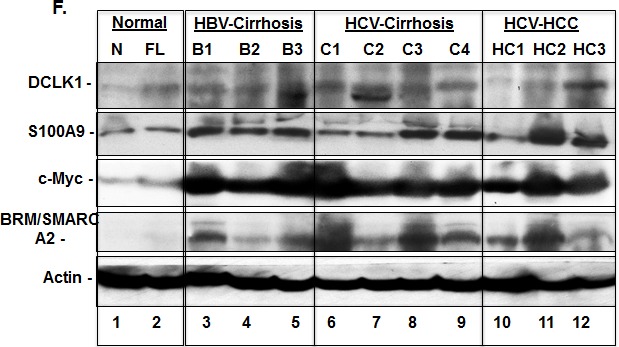
DCLK1 overexpression correlates with increased levels of S100A9, c-Myc and BRM/SMARCA2 in patients with chronic liver diseases and HCC **A.** Immunohistochemical staining was carried out to evaluate DCLK1 and S100A9 expression in different cell populations of liver tissues derived from HCV-positive patients. The composite scores (intensity x percent positive cells) for each cell types were evaluated. Results are expressed as average ± SEM. **B., C., D.** Examples of immunohistochemical staining of human liver tissues: normal adult **B.**, HCV-positive patients **C., D.** with cirrhosis. **C.**, a white male nonresponsive to IFN-Ribavirin treatment; **D.**, an African American male patients with cirrhosis. Arrows (red in D) indicate membrane staining of S100A9 in hepatocytes adjacent to the inflammatory regions. **E.** Co-staining of liver tissues of patient with cirrhosis for CD20 (red) and DCLK1 (Brown) showing DCLK1-positive B lymphocytes (right panel). Two positive cells for both markers are highlighted with one and two arrows and shown at high magnification (60x, left panel). **F.** Western blot analysis of total lysates (30 μg) prepared from patient's liver tissues. *N*, normal liver; *FL*, fatty liver. *B1-B3*, hepatitis B virus (HBV)-positive patients with cirrhosis; *C* and *HC* are samples of cirrhosis and hepatocellular carcinoma respectively from chronic HCV-positive patients.

Immunohistochemical staining was performed on normal liver (6 samples) and liver tissues from HCV-positive patients with cirrhosis (16 samples) or HCC (10 samples). The composite scores that account for the staining intensities and the number of positive cells in the liver are shown in Fig 2A for normal and patients with cirrhosis. In normal liver all the cell subtypes were negative for DCLK1. Only Kupffer cells (resident macrophages) were S100A9-positive. In contrast, epithelial and stromal cells, Kupffer cells, lymphocytes, and bile ducts showed expression of DCLK1 and S100A9 in cirrhosis. Interestingly, increased DCLK1 expression correlated with S100A9 for the most cell-types in HCV-positive cirrhosis.

Lymphoid aggregates/follicles and internodular septa are considered hallmarks of HCV-induced chronic liver disease. Similar aggregates were primarily composed of B and T cells as revealed by their respective markers. The aggregates extensively stained for both DCLK1 and S100A9 (Figures 2C and Supplemental S3) whereas normal liver lacked such staining (Figure 2B). In addition to cytoplasmic staining of hepatocytes and stromal cells in a different patient liver tissues, we also noticed S100A9 staining in hepatocyte membranes in areas adjacent to inflammatory/septal regions (Figure 2D, red arrows). Co-staining with CD20 and DCLK1 suggested that certain B lymphocytes expressed DCLK1 (Figure 2E). It is known that lymphocytes are susceptible to HCV infection and support HCV RNA replication [37]. It is possible that DCLK1 expression in these cells may be induced by HCV.

The expression of DCLK1 in liver tissues and its relationship to S100A9, c-Myc, and BRM for HBV- and HCV-positive patients was determined by Western blot (Figure 2F, lanes 3-12). Most cases with cirrhosis and HCC showed higher expression of all of these proteins compared to normal liver (lane 1). Liver biopsies showing steatosis but no evidence of cirrhosis or HCC also showed elevated DCLK1. However, there was no increase in S100A9, c-Myc, or SMARCA2. The C1 and C2 samples (lanes 6 and 7) had nearly normal levels of S100A9 although all other protein levels were elevated. This may have been due to their known history of immunosuppressive drug use (i.e., prednisone).

### DCLK1 levels correlate with activation of inflammatory cascade

**Figure 3:**
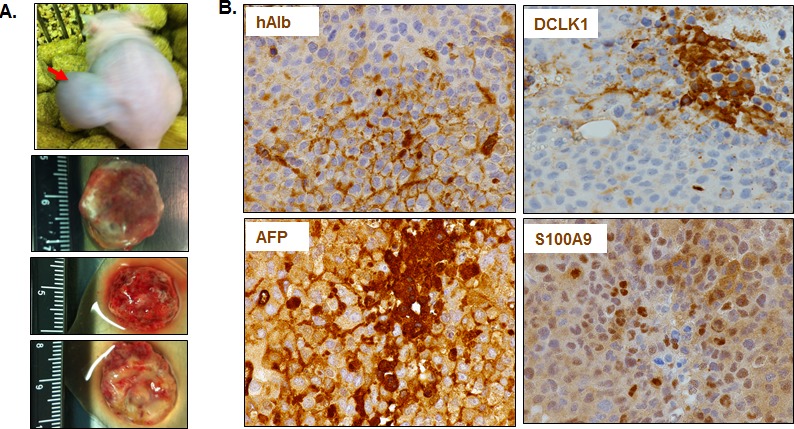

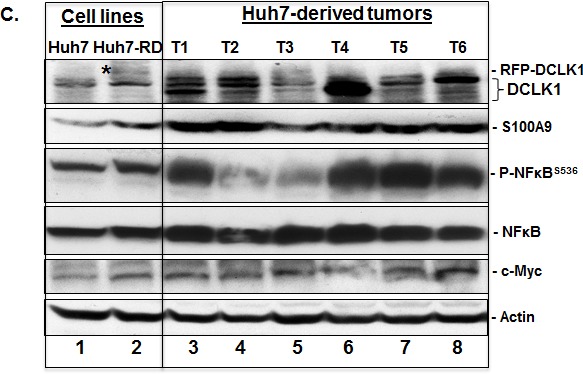
DCLK1 overexpression correlates with the activation of S100A9-NFκB inflammatory pathway in a hepatoma mouse xenografts model **A.** An example of Huh7 hepatoma cells-derived tumor within the flank of a nude athymic mouse. Three representative excised tumors from other mice are also shown. **B.** Representative results showing immunohistochemical staining of the tumors for human albumin (hAlb), DCLK1, α-fetoprotein (AFP) and S100A9. **C.** DCLK1 overexpression correlates with enhanced S100A9 expression, activation of inflammatory pathway indicated by extensive S536 phosphorylation of NFκB, and increase in c-Myc levels in the Huh7-derived tumors. One million Huh7 cells were transplanted into the flanks of nude/SCID mice. Western blot was carried out in total lysates prepared from six Huh7-derived tumors (T1 through T6, lanes 3-8). The levels were compared with the corresponding protein expression levels in the parent Huh7 cell lysates (lane 1) and Huh7-overexpressing human RFP-DCLK1 (Huh-RD, lane 2). Protein band indicated with a star in lane 2 represents recombinant RFP-DCLK1.

We transplanted one million Huh7 cells into the flanks of immunodeficient mice at each site and 7 of 8 transplanted sites developed into tumors (Figure 3A, only 3 tumors shown here). All collected tumors showed expression of human albumin by immunohistochemical staining, suggesting that these tumors had originated from transplanted cells (Figure 3B). Both sporadic clustered cells in certain areas as well as scattered individual cells showed intense staining for DCLK1, α-fetoprotein (AFP) and S100A9. These findings are indicative of aggressive tumor phenotypes as AFP marks hepatoblasts. Western blot analysis suggests that the majority of tumors had higher DCLK1 levels (Figure 3C, lanes 3-8) than transplanted Huh7 cells (lane 1). The increase in DCLK1 correlated with enhanced NFκB activation as assessed by p-NFκB^S536^ levels. Similar observations were made for S100A9 and c-Myc in most tumors. The multiple banding observed for DCLK1 is most likely due to varying phosphorylation status of the protein or alternatively spliced variants [38]. Similarly, Huh7 cells overexpressing recombinant human DCLK1 (Huh7-RD, lane 2) also showed a modest increase in the S100A9, c-Myc, and p-NFκB^S536^ (lane 2) compared to the control (lane 1).

### DCLK1 regulates the calprotectin subunit S100A9

**Figure 4:**
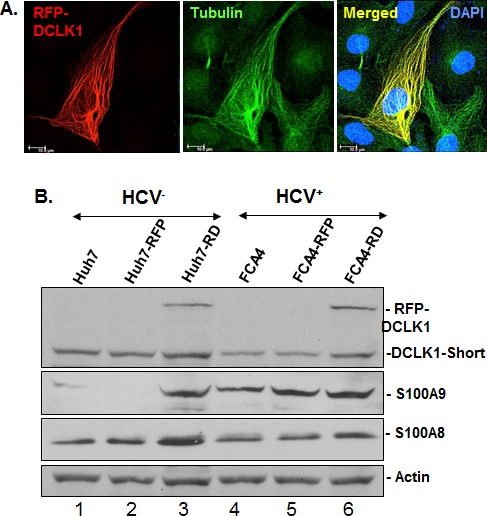

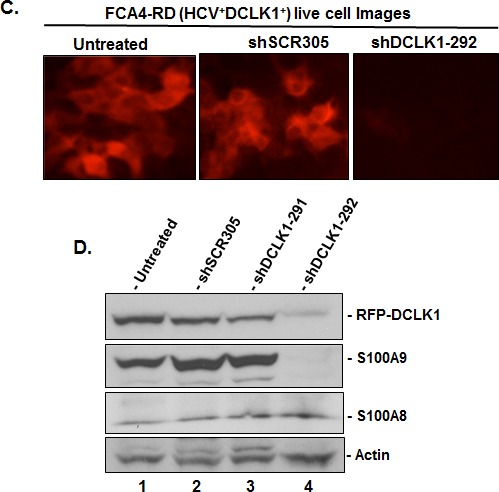
DCLK1 overexpression and HCV induces calprotectin subunit S100A9 **A.** Confocal microscopy for cellular localization of the recombinant RFP-DCLK1 (red) in FCA4-RD cells and its colocalization with α-tubulin (green) that was stained with antibodies. Blue, nucleus. **B.** The florescence-activated cell sorting (FACS)-enriched Huh7-RD (RFP-DCLK1 overexpressing Huh7 cells, lane 3) or FCA4-RD cells (lane 6) were subjected to Western blot. In parallel, corresponding control cell lines expressing RFP alone (lanes 2, 5) or the corresponding parent cell lines (lanes 1, 4) were analyzed. The anti-DCLK1 antibodies (ab109029) used here efficiently detect RFP-DCLK1 and DCLK1-short form (47 KDa). **C.** shRNA-led knockdown of DCLK1 abrogates S100A9 overexpression in FCA4-RD cells. FACS-sorted FCA4-RD cells were infected with lentiviruses expressing scrambled shRNA (shSCR305) or two shRNA against DCLK1; an inefficient shDCLK1-291 and a highly potent shDCLK1-292 for targeting DCLK1. Live cell mages of FCA4-RD showing RFP-DCLK1 expression (red) in control (no exogenous shRNA, untreated) or cells expressing scrambled (shSCR305) or anti-DCLK1 shRNA-292. **D.** Western blot analysis of the total cell lysates for DCLK1, S100A8 and S100A9 expression in the control (lane 1) or cells expressing scrambled (lane 2), ineffective shDCLK1-291 (lane 3) and highly effective shDCLK1-291 (lane 4) shRNAs.

The RFP-tagged human DCLK1 (RFP-DCLK1, red) showed colocalization (merged) with microtubules (green) in FCA4-RD cells (Figure 4A) by confocal microscopy whereas RFP alone showed only random cytoplasmic distribution (not shown). Live imaging exhibited dynamic distribution of RFP-DCLK1 along with microtubule filaments and accumulation at spindle poles during cell division (not shown). These results suggest that recombinant DCLK1 preserves its microtubule binding activities in the cells. Huh7 and FCA4 cell lines expressing RFP or RFP-DCLK1 isolated by FACS and cultured in regular media were subjected to Western blot analysis. Total lysates of RFP-DCLK1-expressing Huh7 cells (Huh7-RD) showed higher S100A9 protein (Figure 4B, lane 3) compared to corresponding controls (lanes 1, 2). The anti-DCLK1 antibodies used for probing recognized recombinant and the short form of DCLK1 (upper panel). In FCA4 cells, HCV replicons alone caused a modest increase in S100A9 but not S100A8 (lane 4). Relatively higher expression of S100A9 was also seen in the FCA4-RD line (lane 6). To confirm if S100A9 expression was regulated by DCLK1, we prepared FCA4-RD cells expressing scrambled shRNA (shSCR305) or two shRNAs designed to target DCLK1 (shDCLK1) mRNAs. In a preliminary experiment, we found that only shDCLK1-292 but not shDCLK1291 was effective in knocking down DCLK1 expression within 48-72 hours of induction (not shown). As shown in Figure 4C, ShDCLK1-292 but not scrambled shRNA, reduced RFP-DCLK1 expression in live cells. This observation was confirmed by Western blot analysis (Figure 4D). DCLK1 knockdown specifically inhibited S100A9 but not S100A8 expression (lane 4). Both overexpression and knockdown assays suggest that DCLK1 functions upstream of S100A9 and regulates expression of the S100A9 subunit of calprotectin.

### DCLK1 overexpression enhances migration of hepatoma cells

**Figure 5:**
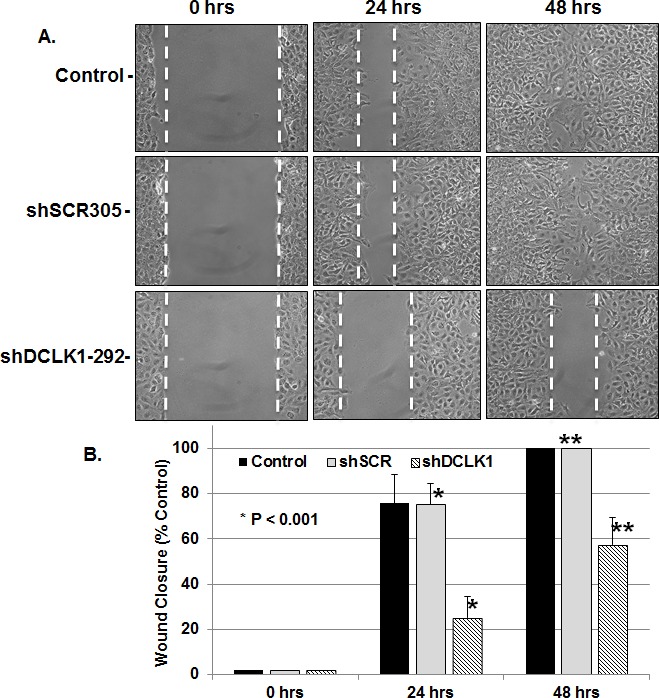

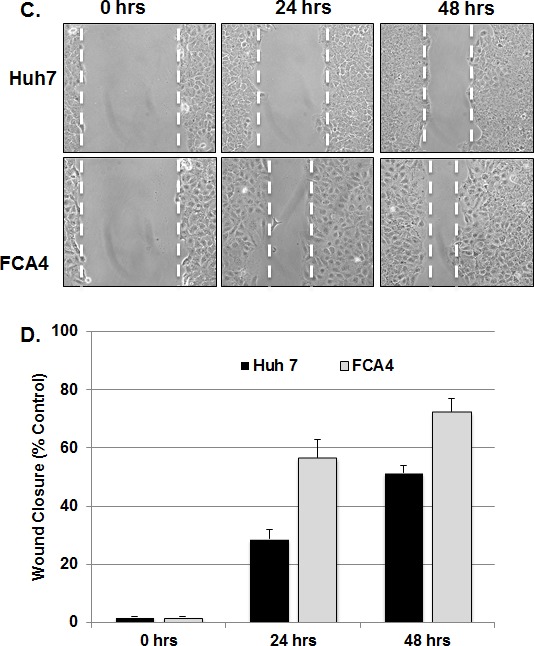
DCLK1 knockdown results in inhibition of FCA4-RD cell migration **A.** Representative images of wound healing assay at 0, 24 and 48 hr after the induction of scrambled shSCR305 or anti-DCLK1 shDCLK1-292. Control, FCA4-RD cells. **B.** Quantitative representation of results obtained for three separate wound healing assays and statistical analysis. The graphs shown are mean ± SD. Stars, comparison of the samples with the control. **C** and **D**. Wound healing assay for the parental cell line (Huh7) and FCA4 cells.

Collective cell migration has been suggested to play essential roles in invasion and metastasis of malignant tumors [39]. DCLK1 has been shown to promote neuronal migration [40], polymerization of microtubules [33], epithelial-mesenchymal transition [41] and expression of S100A9 (Figure 4). Interestingly, S100A9 protein also promotes microtubules reorganization [42], cell migration and invasion through p38 MAPK-dependent NFκB activation [43]. Thus, targeting DCLK1 alone should diminish migratory abilities of FCA4-RD cells. To examine this possibility, FCA4-RD cells expressing scrambled shRNA (shSCR305) or anti-DCLK1 shRNA (shRNA-292) were subjected to the wound-healing assay and compared to the extent of wound closure with the parent FCA4-RD cells (control). As shown in Figure 5, the rate of wound closure was much slower (4 times less at 24 hr) in the cells expressing shRNA-292 than the controls (Figures 5A and 5B). Both shSCR305 and untreated control exhibited similar wound healing patterns. It is known that the wound healing process begins when the cells polarize toward the wound, initiate protrusion, migrate and close the wound area. These and other results (Figure 4) provide proof-of-concept data that the DCLK1 knockdown is possible in the DCLK1-overexpressing hepatoma cells and such treatment will eventually halt migratory abilities of the tumor cells. The migratory ability of FCA4-RD cells were compared with that of the parent Huh7 and FCA4 cells (Figure 5C). The results clearly show that migration of FCA4 (~75%) is higher than parental Huh7 cell line (~55%) but less than FCA4-RD (HCV^+^DCLK1^+^) cells (~100%) within 48 hr (Figures 5B and 5D).

### Normal human hepatocytes express DCLK1 and acquire stem-like properties in Matrigel matrix

**Figure 6:**
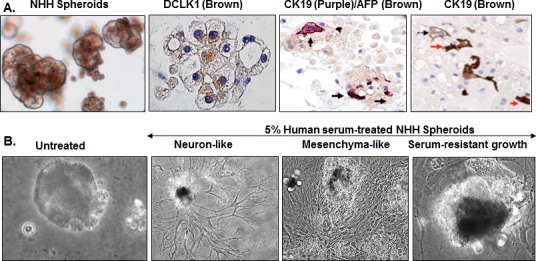
Cellular plasticity and transforming potential of normal human hepatocytes (NHHs) expressing endogenous DCLK1 **A.** Hepatocytes preparations were made from adult human healthy liver and cultured in Matrigel for 3-6 weeks. Induction of perinuclear DCLK1 expression in normal human hepatocytes (NHHs) within the spheroids and their differentiation into multiple cell-lineages are shown by immunohistochemical staining of the spheroids for DCLK1 and α-fetoprotein/cytokeratin 19 (AFP/CK19) co-expression or cytoplasmic CK19 (both mark stem-like progenitor cells). **B.** Human serum (5%)-induced differentiation and morphological changes in the NHH-spheroids (bright-field images). Representative morphological patterns for differentiated (e.g. neurons, mesenchymal cells) or serum-resistant (extreme right panel) of the spheroids are shown.

We observed that normal liver parenchyma lacks DCLK1 staining and DCLK1 bands are barely seen in the Western blot analysis. However, it is expressed above the baseline in non-HCV cases such as NASH and in chronic hepatitis B (Figure 2) suggesting that DCLK1 can also be induced by other factors. Because DCLK1 is considered as a CSC marker, we cultured DCLK1-negative normal human hepatocytes (NHHs) preparations [44] in Matrigel that is known to contain a number of growth factors and supports growths of stem-like cells. The resulting spheroids were analyzed after 4 weeks for expression of DCLK1 and hepatic markers. The spheroids exhibited eccentric, perinuclear staining of DCLK1 in most cells (Figure 6A) except in the asymmetric ductules. These spheroids also exhibited heterogeneous hepatic cell lineages such as AFP^+^ hepatoblasts and progenitor/stem-like cells marked by cytoplasmic CK19 staining or AFP/CK19 co-staining (Figure 6A, shown with arrows). A number of cells in the spheroids were negative for these markers but a few cells were positive for human albumin (not shown). Stimulation of the spheroids with 5% human serum resulted in either morphological changes suggesting differentiation into neuronal-like and mesenchymal-like cells or showed resistance to serum-induced changes (Figure 6B). These findings fit very well with recently proposed model that hepatocytes possess significant plasticity [45], which might be controlled by the DCLK1 expression followed by differentiation into multiple subtypes.

## DISCUSSION

We previously demonstrated that sustained HCV replicon expression in hepatoma cell lines is directly linked to the gain of cancer stem cell (CSC)-like properties and overexpression of CSC markers (DCLK1, Lgr5, CD133, c-Myc) [16]. Because the onset of cirrhosis and HCC occurs during chronic HCV infection, an increase in DCLK1 is not observed in an acute infection model. For this reason, we used a well-characterized FCA4 cell line that represents heterogeneous hepatoma cell populations and supports persistent replication of the HCV RNAs. This cell line and cells derived from it partially mimic intracellular changes that might occur during chronic infection. Thus the observed effects in these cells are primarily attributable to intracellular changes in response to chronic viral replication. The findings from these cell lines were corroborated using biopsies from livers of patients with HCV and HBV infections, normal human hepatocytes-derived spheroids, and *in vivo* studies in murine xenografts. During transcriptome analysis, we observed that DCLK1 overexpression results in significant increase in S100A9 mRNA level (18.29±0.0002, Table S1), which was validated by Western blot. Because chronic inflammation associated with HCV infection is considered a major contributor to cirrhosis and the development of HCC, the transcriptome data provided legitimate basis to investigate the relationship between DCLK1 and S100A9 in the context of HCV infection.

Our studies show control of DCLK1 over S100A9 expression. The S100A9 protein forms physiologically relevant S100A8/A9 heterodimer (MRP8/14 or calprotectin) and multimer complexes [27], activates NFκB, and increases phosphorylation of MAP kinases [46]. Elevated levels of S100A8/A9 heterodimers have been reported in inflammatory diseases, cancers, and autoimmunity [26]. It is also a secretory protein and interacts with the cell surface toll-like receptor 4 (TLR4). During validation of the transcriptome data (Figure 1F), we noticed that S100A9 in FCA4-RD lysates was only modestly higher (lane 4) than its corresponding control (lane 3). It will be prudent to study in future if this is due to S100A9 secretion by FCA4-RD cells into the media. The S100A9-TLR4 interaction can facilitate multiple downstream signaling processes including NFκB activation. We noticed that a large number of stromal cells and hepatocyte-like cells within the regenerative nodules exhibit extensive expression of DCLK1 and membrane bound S100A9. Such responses likely stimulate additional transcription of S100A9, S100A8/A9 multimerization, and polymerization of microtubules that induce cellular migration [47]. In addition, clinical trials with the S100A9 inhibitor, tasquinimod (TasQ), that disrupts S100A9-TLR4 interactions has shown limited efficacy against castrate-resistant prostate cancer [48]. During Western blot assays, we observed both monomers (14 kDa) and multimers (~49 kDa) of S100A9 protein bands were significantly reduced following DCLK1 knockdown by siRNAs. Because DCLK1 directly controls S100A9 expression, our studies suggest that a combination of an anti-DCLK1 drug with TasQ would be a more rational treatment approach for these tumors.

**Figure 7:**
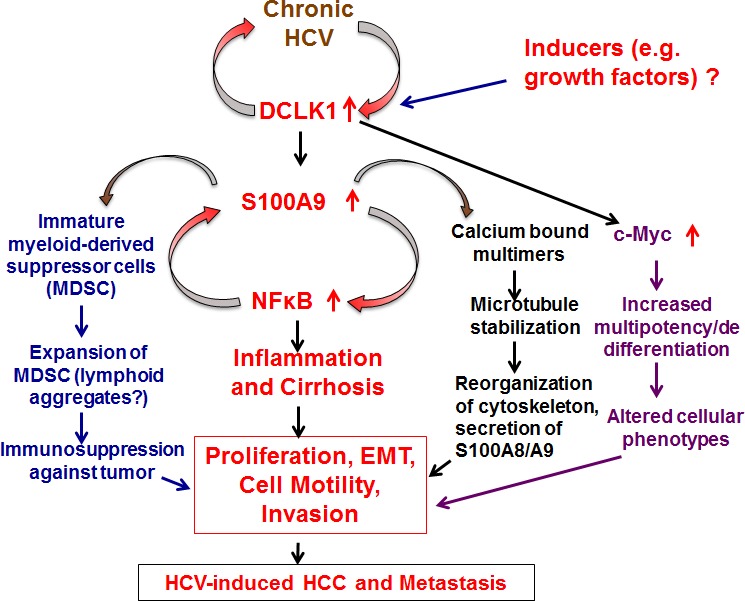
Schematic presentation of DCLK1 signaling and two distinct feed-forward-like regulations during HCV-induced hepatocarcinogenesis DCLK1 induced by HCV or other factors (e.g. growth factors, Figure 6) is likely to affect downstream multiple pathways such as inflammation, microtubule cytoskeleton reorganization, immunosuppression and oncogenic stimulus via c-Myc. The studies presented here were primarily focused on the inflammatory cascades. Additional studies will be needed to delineate how cellular migration (Figure 5) occurs via DCLK1-S100A9-induced cytoskeleton reorganization and secretion of S100A9 external microenvironments of the infected hepatocytes. The DCLK1-controlled regulation of c-Myc has also been shown previously in other cell lines [58].

Another consequence of DCLK1 overexpression may relate to the maturation of myeloid-derived suppressor cells (MDSC) and reorganization of cytoskeletons DCLK1-S100A9-microtubule modules (Figure 7). MDSCs are heterogeneous group of activated myeloid progenitor and immature myeloid cells (IMCs). IMCs rapidly differentiate into mature myeloid cells under normal physiological conditions. However, colony stimulating cytokines, VEGF, S100A8/A9, and NFκB promote accumulation and activation of MDSC [49]. These cells suppress the adaptive immune response against tumors by blocking the functions of CD4^+^ and CD8^+^ T cells [50]. Thus, DCLK1 may exert influence on immune suppression, tumor microenvironment, and tumor-stromal interactions by modulating S100A9 and NFκB activities.

One histologic characteristic of chronic HCV infection is the presence of lymphoid aggregates in the liver that are composed of clonally restricted B cells. The potential mechanism(s) by which chronic HCV infection predisposes patients to type II mixed cryoglobulinemia and premalignant/malignant B-cell lymphoproliferations are poorly understood. We demonstrated that portal inflammation with dense lymphoid aggregates in HCV patients are positive for DCLK1 and enriched in CD20^+^ B cells. CD20 is expressed on the cell surface of all B cells except early pro-B lymphocytes and plasma cells. Analysis of HCV lymphotropism suggests that CD20^+^ cells are the major lymphocyte repertoire that supports HCV replication [51]. The activation of proinflammatory-tumorigenic stimuli via a DCLK1 pathway may collectively explain the association of chronic HCV infection with mixed cryoglobulinemia and B cell malignancies (Figures 2C and 7).

The HCC also occurs in patients who are not infected with HCV or HBV. DCLK1 expression is extremely low in normal human liver parenchyma (Figures 2B and 2F), and in normal cultured hepatocytes on collagen I-coated culture plates [44]. In contrast, hepatocytes that are cultured as spheroids in Matrigel exhibit perinuclear DCLK1 expression in most cells. Matrigel provides at least many growth factors (e.g. TGF-β, EGF, IGF, and FGF) and abundant extracellular matrix (ECM). Under these conditions, we observed hyperplasticity and transforming potential of normal adult hepatocytes, which explains liver diseases in metabolic disorders that are independent of viral infection. (e.g. NASH). Secondly, our results highlight functional heterogeneity of the spheroids. Upon stimulation with human serum, spheroids differentiated into multiple cell-lineages, including neuron-like cells, with a few growing into larger undifferentiated spheroids. Lineage-tracing experiments should help confirm whether CK19^+^, DCLK1^+^, AFP^+^, AFP^+^CK19^+^ cells in these spheroids (Figure 6B) are directly derived from DCLK1^+^ hepatocytes.

Among differentially expressed genes, BRM (SMARCA2) showed a direct correlation with enhanced expression of DCLK1, c-Myc and S100A9 in FCA4-RD cells as well as in the liver of HBV- and HCV-positive patients. BRM and BRG1 (SMARCA4) are paralogs and only one of these proteins is incorporated into the SWI/SNF chromatin remodeling complex as ATPase subunit [52]. In cancer cells, BRG1 is downregulated or inactivated due to mutations [53, 54]. As a result, the nonessential BRM is incorporated into the SWI/SNF complexes causing altered SWI/SNF functions that promote tumor development. It has been shown that BRM null/BRG1-positive mice lack the CSC marker CD44 expression [55]. These observations indicate a possible role of DCLK1 in the regulation of CD44 via BRM.

We observed during our studies that Huh7 cells-derived tumor growth can be inhibited in a murine model by administering Poly Lactic-co-Glycolic Acid (PLGA) nanoparticles (NPs) encapsulated anti-DCLK1 siRNAs inhibited (communicated elsewhere). In addition, we have also presented evidence that DCLK1 overexpression alters expression of an array of genes, which are involved in inflammation and tumor growth (Figures 1A and 1D, Table S1). Further investigations will reveal how DCLK1 regulates signaling network involving these proteins in normal and disease conditions. Our data underscores the importance of targeting DCLK1 not only in chronic liver diseases and HCC but also tumors of intestine, colon and pancreas where DCLK1 has been shown to be overexpressed [41, 56], In summary, the studies describe a novel feed-forward-like loop of DCLK1 signaling network that appears to control inflammation and neoplastic transformation during chronic liver diseases. It also highlights DCLK1 as an important target for the treatment of inflammatory and neoplastic diseases.

## MATERIALS AND METHODS

### Human liver tissues and cell culture

The normal and diseased human liver tissues were obtained from the Liver Tissue Cell Distribution System (Minnesota). The cryopreserved normal human hepatocytes (NHHs) were purchased from BD Biosciences. This study was exempted from IRB review after institutional IRB review (IRB#: 3405).

Hepatocytes were cultured in Hepato-STIM Hepatocyte Defined Medium (BD Biosciences) supplemented EGF (10 ng/ml), 2 mM L-glutamine and 1X antibiotic-antimycotic on collagen cover slips or in Matrigel plates. The FCA4 cells are derived from Huh7 hepatoma cell line and express a G418-resistant HCV-1b subgenomic replicon encoding NS3 through NS5A proteins and has been characterized previously [16, 36]. The real-time reverse transcription-PCR analyses were carried out using total RNA as described earlier [16]. The mRNA levels were expressed as fold change relative to control with ± SEM value. Total lysates of culture cells were prepared according to the protocol described for Pierce M-PER cell lysis buffer (Thermo-Fisher) for Western blot. The immunohistochemistry (IHC), confocal Microscopy and florescence-activated cell sorting (FACS) were carried out using standard methods.

### Tumor xenografts in murine model

The studies reported here with mice were approved (Approval Protocol # 12-041) and supervised by the University of Oklahoma Health Sciences Center (OUHSC) Institutional Animal Care and Use Committee (IACUC) which adheres to the PHS Policy IV.B.3.

Athymic nude Balb/c mice were purchased from Jackson Laboratory and housed in pathogen-free conditions. One million Huh7 cells were washed with PBS three times, resuspended in the same buffer, and injected subcutaneously into the dorsal flanks of 4–6 week old mice. Tumors were measured with calipers and the volumes were calculated using formula: 0.5 X (length X width^2^). Four weeks later, one small (0.36 cm^3^) and six large (average 1.5 cm^3^ to 4 cm^3^) tumors were resected from the animals. The pieces of the tumors were preserved in 5% formalin for immunohistochemistry, stored at −80°C in RNase inhibitors for real-time PCR and directly frozen for Western blot analyses. Three additional animals also received similar amounts of PBS but did not develop tumor during the experiment. The cancer-related molecular markers in these tumor lysates were analyzed by Western blot. The lysates of the tumor or liver tissues were prepared using standard RIPA buffer procedure. Twenty-five micrograms of the lysates were subjected to gradient SDS-PAGE (8%–12%) and the protein bands were transferred onto a PVDF membrane for detection of the protein bands. The intensity of actin band (42-kD) in each lane was considered as a loading control.

### Immunohistochemistry (IHC)

The IHC was performed according to manufacturer's protocol using Leica Bond-III^TM^ Polymer Refine Detection system (DS 9800). The paraffin-embedded tissues were sectioned and mounted on positively charged slides. The slides were dried overnight at room temperature and incubated at 60^°^C for 45 minutes followed by deparaffinization and rehydration in an automated Multistainer (Leica ST5020). These slides were transferred to the Leica Bond-III^TM^, treated for target retrieval at 100°C for 20 minutes in a retrieval solution. Endogenous peroxidase was blocked using peroxidase-blocking reagent, followed by the primary antibody incubation for 60 minutes. The IgG-linker and/or Poly-HRP IgG reagents were used as secondary antibodies. Detection was done using 3, 3′-diaminobenzidine tetrahydrochloride (DAB) as a chromogen and counterstained with hematoxylin. Each staining was carried out with appropriate negative and positive e controls. For double staining, Leica Bond-IIITM Polymer Refine Detection system (DS9800) and Leica Bond-III^TM^ Refine Red Detection system (DS9390) were used sequentially.

The slides and tissue arrays were subjected to immunohistochemical staining using anti-DCLK1 antibodies (ab31704, Abcam) and S100A9 (AJ1507a, Abgent). The scoring of DCLK1 or S100A9 staining was carried out based on two parameters (staining intensity and amount of tissue involved). The intensity was measured and scored from 0 – 4 where 0= no staining, 1= very weak, 2= weak, 3= moderate and 4= strong. The amount of tissue involved was scored from 0 – 4, based on the percent of involvement; no tissue involved = 0, 1= <10%, 2= 10%-30%, 3= 31%-60%, and 4= >60%. Finally, the composite scores were generated by multiplying the intensity scores with the tissue involvement scores, and the results were plotted for different cell types.

### Real-time RT-PCR

The real-time reverse transcription-PCR analyses were carried out using total RNA as described earlier [16]. Total RNAs were isolated from treated and untreated tumors or cultured cell using RNeasy isolation kit (Qiagen). The RNA samples were analyzed for purity/integrity and subjected to reverse transcription with Superscript II and random hexanucleotide primers (Invitrogen). In the subsequent step, the cDNAs were used as templates to perform real-time PCR by SYBR chemistry method (SYBR^®^ Green I; Molecular Probes). The target (HCV, DCLK1) and control (actin) RNAs were amplified using Jumpstart Taq. The crossing threshold values assessed by the real-time PCR were evaluated for the transcripts. Actin mRNA level in each sample served as an internal control. The mRNA levels were expressed as fold change relative to control with ± SEM value.

### DCLK1 overexpression

The red fluorescence protein (RFP) coding sequences were cloned in-frame at the 5′ end of DCLK1 (NM_004734) ORF to generate RFP-DCLK1 cassette in pENTR-DsRedEx2 vector (Addgene). Using clonase, the cassette was transferred to pLenti-CMV-PURO-Dest plasmid to generate pLenti-RFP-DCLK1 plasmid vector. The expression plasmid was packaged into lentivirus by transient plasmid transfection of 293T cells together with the three helper plasmids (pMD2.G, pMDL/RRE g/p, and pRSV-REV). The viral particles in the supernatants were concentrated. Following infection, the cells were selected for 7-10 days in the presence of puromycin (10 μg/ml). The resistant cells were further grown under normal culture conditions without puromycin. A control expression vector (pLenti-RFP) expresses only RFP and was packaged into viruses as described above to develop control cell lines. Total lysates were prepared from cultured cells using M-PER lysis buffer (Pierce) and Western blots were carried out by chemiluminescence method (GE Healthcare). The primary antibodies against HCV NS5B, DCLK1 and actin (all purchased from Abcam) were used for the detection of respective protein bands. The band intensities were calculated using Gelquant software to evaluate target protein to actin ratios.

### Immunofluorescence, confocal microscopy and florescence-activated cell sorting (FACS)

Cells grown on glass cover-slips (VWR) were rinsed briefly in phosphate-buffered saline (PBS), fixed in 4% paraformaldehyde in PBS pH 7.4 for 20 min at room temperature, washed twice with ice cold PBS and permeabilized in ice-cold acetone. Cells were incubated with blocking buffer (10% serum, 0.01 % Triton X-100, in PBS, pH 7.4) for 1 hour, washed with PBS and treated with anti-DCLK1 (Abcam) and/or anti-α-tubulin (Santa Cruz) antibodies in PBS-T containing 1% BSA for 1-2 hr at room temperature or overnight at 4°C. After thorough washing with PBS-T, cover-slips were incubated in appropriate AlexaFluor conjugated secondary antibodies. The nuclei were counterstained with DAPI (0.1-1 μg/ml PBS). The cover-slips were mounted on microscope slides in ProLong Gold antifade reagent (Invitrogen) for detection of Immunofluorescence using Nikon 80i or Leica TCS NT (for confocal microscopy).

The RFP and RFP-DCLK1 expressing cultured cells were trypsinized, resuspended in regular media and centrifuged. The cell pellets were resuspended into Dulbecco's phosphate buffer saline (DPBS, without Ca^++^/Mg^++^) containing fetal bovine serum (1%). The RFP-expressing cells were enriched by FACS method and the sorted cells were re-plated in the regular DMEM media.

### Genome-wide RNA analysis in DCLK1 overexpressing cells

Total RNAs from cultured cells were isolated using RNeasy Plus mini kit (Qiagen) and RNA qualities for each sample were analyzed using the Agilent Bioanalyzer 2100 and RNA nano total RNA chips. An RNA integrity number (RIN) of >6 is considered as a good RNA preparation. The cDNA library was constructed from 1.0 μg of total RNA using the Illumina TruSeq total RNA V2 kit and established protocols. Three samples per run were used for collecting sufficient data to determine the most highly expressed genes from each sample. A minimum of 30 million 250 bp paired end sequencing reads were collected on each run and data were analyzed using Genesifter software (Perkin Elmer). Raw data for each sample were mapped to the most recent human genome build for identification of both exon and intergenic regions. Tertiary bioinformatics analysis (pairwise comparison) of the expression results were performed and information from both KEGGs and Gene Ontology databases were analyzed for identification of mRNAs that are differentially expressed at a significant level. The protein expression levels for selected genes were validated using Western blot.

### Generation of lentiviruses and inducible-shRNA expression system

The DCLK1-targeting shRNAs were generated as described previously [57]. Sense and anti-sense oligos (IDT) were annealed and ligated into Bspm1 cut pEN_TTRmiRc2 (Addgene; Cambridge, MA) to generate pEN_TTR miRshDCLK1. Then pEN_TTR miRshDCLK1 and pSLIK Hygro (Addgene; Cambridge, MA) were *in vitro* recombined using Gateway LR Clonase II (Invitrogen, Grand Island, NY) to create pSLIK TTR miRshDCLK1-hygro. Lentiviruses were prepared by the PEI transfection of HEK293T cells plated on poly-L-lysine (Sigma; St. Louis, MO) coated plates with 10 μg transfer vector, 2.5 μg pMD2.G, 2.5 μg pMDL/RRE g/p, 2.5 μg pRSV-REV per 10cm plate. Cell supernatant was collected 48 h and 72 h post-transfection, filtered with a 0.45 micron MES filter, and precipitated using polyethylene glycol. Virus was resuspended in DMEM and added to cells in the presence of 8 μg/ml of polybrene (Sigma; St. Louis, MO) overnight. The shRNAs were induced for expression by adding doxycycline (1 μg/ml media). For wound-healing assay, the FCA4-RD cells (10,000) were seeded in the wells of 12-well plates to ~90% confluency and wounds were scratched using a sterile pipette tip 3–6 h post seeding. The same spots were photographed under phase contrast microscopy at various time points to monitor cell migration/proliferation. The experiment was repeated three times and quantitative analysis was carried out for the extent of wound healing at various time points.

## SUPPLEMENTARY FIGURES AND TABLES


