# Supplementary material for: Inflammatory and oncogenic roles of a tumor stem cell marker doublecortin-like kinase (DCLK1) in virus-induced chronic liver diseases
Source: Oncotarget. 2015 Apr 29;6(24):20327–44. doi: 10.18632/oncotarget.3972 (PMC4653008; doi:10.18632/oncotarget.3972)
Supplement: Supplementary file 1 [file oncotarget-06-20327-s001.pdf]

Inflammatory and oncogenic roles of a tumor stem cell marker doublecortin-like kinase (DCLK1) in virus-induced chronic liver diseases

Supplementary Material

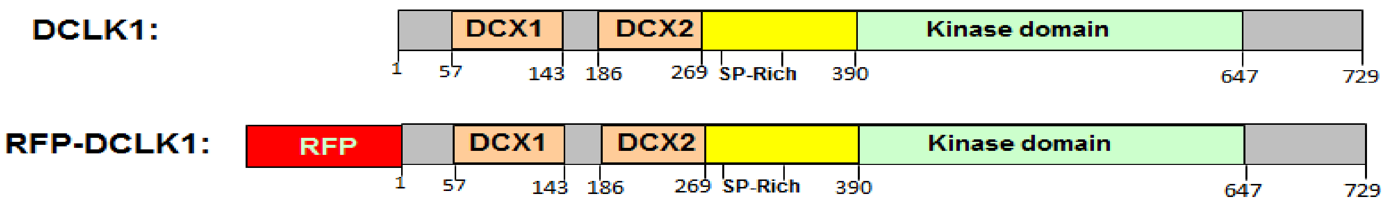

FIG S1 Organization of DCLK1 protein domains. Recombinant DCLK1 tagged with red fluorescence protein (RFP) at the N-terminus (RFP-DCLK1) is expressed in Huh7-RD or FCA4-RD. Two doublecortin domains (DCX1 and DCX2), Ser-Pro rich domains (SP-rich) and a kinase domain are indicated.

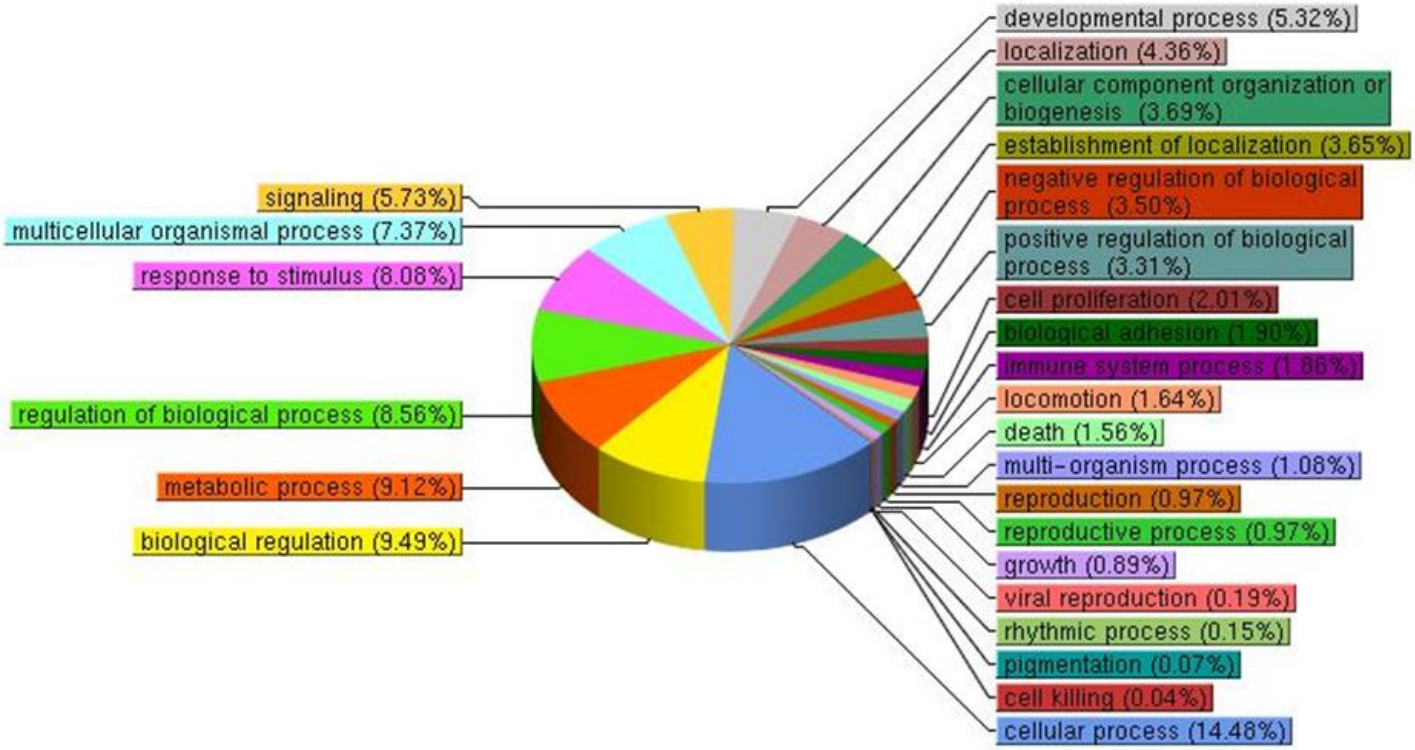

FIG S2 Gene ontology report showing functional distribution of genes influenced by HCV and DCLK1 co-expression.

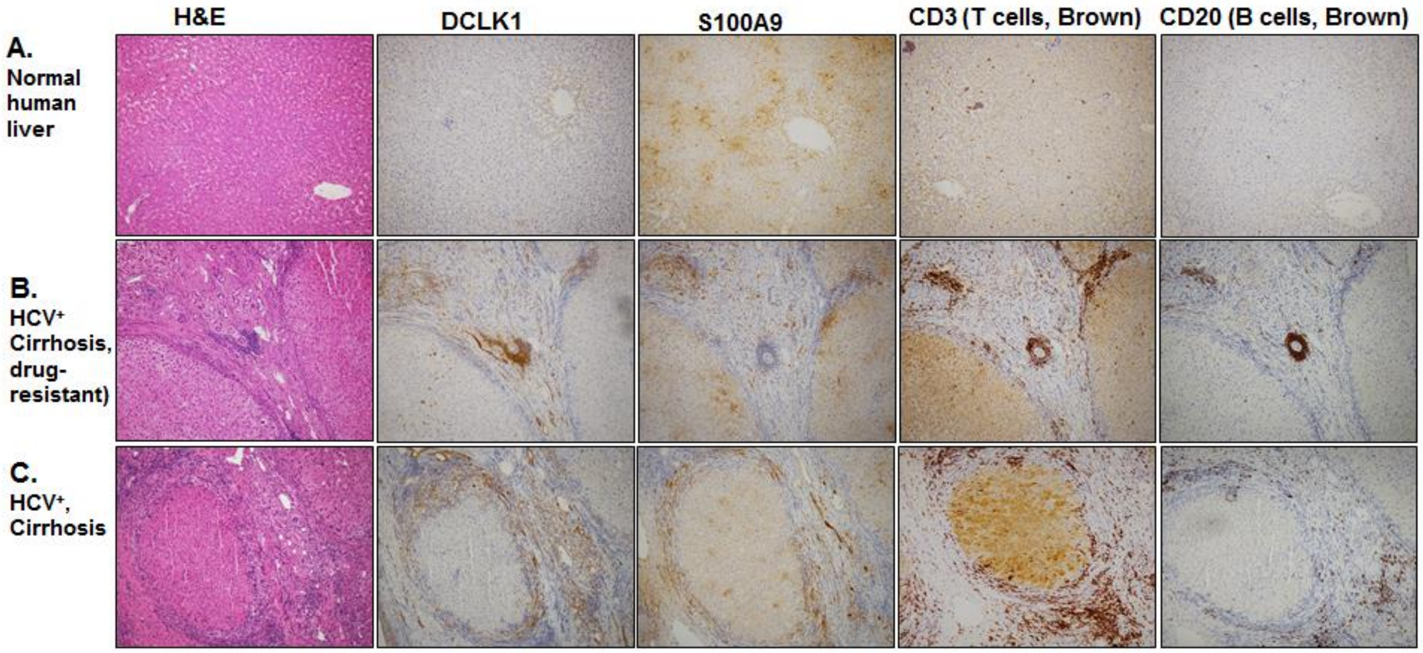

FIG S3 Immunohistochemical staining results for liver tissues derived from HCV-positive patients at lower magnifications to show pattern of staining in larger areas of the liver. Magnification=10x. The Figures 2, B, C, D, are derived from these images but at 60x magnification.

Table S1 DCLK1-responsive genes**.** Summary of differentially expressed genes due to DCLK1 overexpression in Huh7 cells with statistical analysis (*p*-values are shown for each gene).

GENE ID	FOLD INCREASE	ADJUSTED p-VALUE
**DCLK1**	**148.01**	**0.00E+00**
100128460	24.11	6.01E-06
DDT	21.62	2.98E-05
**S100A9**	**18.29**	**2.40E-04**
FGF18	14.97	1.79E-03
TLE6	14.97	1.79E-03
9668	13.3	4.63E-03
OLFML1	13.3	4.63E-03
PHLDA2	13.3	4.63E-03
ESPNP	13.3	4.63E-03
RP11-631M21.2	11.64	1.21E-02
COL8A2	11.64	1.21E-02
FLJ23834	11.64	7.99E-05
CTSK	10.81	1.93E-02
100507111	10.81	1.93E-02
XDH	10.81	1.93E-02
PKD2L1	10.81	1.93E-02
LOC100129195	9.98	3.04E-02
DCDC2B	9.98	3.04E-02
PYY	9.98	3.04E-02
PAQR8	9.98	3.04E-02
100507213	9.98	5.63E-04
SLC6A16	9.98	3.04E-02
100506346	9.15	4.68E-02
GHRL	9.15	4.68E-02
LOC729603	9.15	1.44E-03
ZNF699	9.15	4.68E-02
693224	9.15	4.68E-02
POU3F2	9.15	1.44E-03
EPO	9.15	1.44E-03
SPRY3	8.32	7.24E-02
26816	8.32	7.24E-02
NPTX2	8.32	3.57E-03
LOC100132460	8.32	3.57E-03
LOC100129858	8.32	7.24E-02
LOC642340	8.32	7.24E-02
C3orf42	8.32	7.24E-02
LRRC33	8.32	7.24E-02
LTBP2	8.32	7.24E-02
SLC6A15	8.32	3.57E-03
CRISP3	8.32	7.24E-02
SLC27A5	8.32	7.24E-02
CN5H6.4	8.32	7.24E-02
CXorf22	8.32	3.57E-03
GRB7	8.32	7.24E-02
CDC42EP5	8.32	7.24E-02
IL1RN	8.32	7.24E-02
DUSP26	8.32	7.24E-02
GLDN	8.32	7.24E-02
LOC100130856	7.9	5.55E-03
26784	7.48	8.60E-03
ZNF331	7.48	8.60E-03
CYP2S1	7.48	8.60E-03
LOC653781	7.48	8.60E-03
INHA	7.07	1.34E-02
SULF2	6.86	8.09E-09
PRDM1	6.65	2.04E-02
ZRSR2	6.65	2.04E-02
HEY1	6.65	2.04E-02
PDZRN3	6.65	2.04E-02
DLK1	6.58	2.28E-12
LRP5L	6.24	3.11E-02
HIST1H2AL	6.24	3.11E-02
LFNG	6.1	5.80E-11
LOC219347	5.82	4.61E-02
hCG_2008140	5.82	4.61E-02
100289341	5.82	4.61E-02
FAM108C1	5.82	4.61E-02
VWA5A	5.82	4.61E-02
KLHL26	5.82	4.61E-02
100505945	5.82	4.61E-02
CA5BP	5.82	1.40E-03
ASB2	5.82	4.61E-02
648359	5.82	4.61E-02
TMEM154	5.82	4.61E-02
C9orf117	5.61	2.12E-03
DDN	5.41	3.18E-03
PLAUR	5.41	6.88E-02
HOXD1	5.41	4.25E-02
SLC41A1	5.41	4.28E-02
PCDHB11	5.41	4.31E-02
ZNF788	5.27	4.34E-02
ZNF580	5.2	4.38E-02
GUCY2C	5.16	4.41E-02

GENE ID	FOLD DECREASE	ADJUSTED p-VALUE
MYLK4	21.65	1.02E-04
APBB1IP	16.84	1.47E-03
LAMC2	15.63	2.76E-03
ANXA8	14.43	5.13E-03
LOC202181	12.03	1.78E-02
100507227	12.03	1.78E-02
IL31RA	12.03	1.78E-02
LOC285768	12.03	1.78E-02
100506266	12.03	1.78E-02
FBXO16	12.03	1.78E-02
692093	12.03	1.78E-02
PTPRE	12.03	1.78E-02
SH3TC2	12.03	1.78E-02
BHLHE41	11.42	3.40E-04
CA5B	10.82	6.35E-04
ZNF850P	9.62	2.13E-03
PITPNM3	9.62	2.13E-03
100302692	9.62	2.13E-03
TMEM27	9.02	3.83E-03
344405	8.42	6.83E-03
100506503	8.42	4.28E-04
ZNF442	8.42	6.83E-03
GPLD1	7.82	1.22E-02
GRAMD2	7.22	2.11E-02
C8orf47	7.22	2.11E-02
CCBP2	7.22	2.11E-02
100505616	7.22	2.11E-02
LOC100129250	6.61	3.62E-02
100288069	6.61	3.62E-02
WHAMML1	6.61	3.62E-02
C19orf26	6.31	1.44E-03
286749	6.01	6.07E-02
B3GALT2	6.01	2.67E-06
100505668	6.01	6.07E-02
LRRIQ3	6.01	6.07E-02
IL7	6.01	6.07E-02
401317	6.01	6.07E-02
hCG_1811337	6.01	6.07E-02
100507006	6.01	6.07E-02
100505734	6.01	6.07E-02
LOC100128292	6.01	6.07E-02
BDNF	6.01	6.07E-02
100132101	6.01	6.07E-02
GBP1	5.79	0.00E+00
CCDC80	5.71	1.91E-11
ERAP2	5.41	7.04E-03
FILIP1	5.41	7.04E-03
LYPD6B	5.41	2.56E-05
KCND1	5.41	7.04E-03
UPK3A	5.29	2.33E-03
